# Probiotics in atherosclerosis: mechanisms, efficacy and future directions — a review study

**DOI:** 10.3389/fimmu.2026.1722134

**Published:** 2026-03-25

**Authors:** Yue Cai

**Affiliations:** 1 Yunnan Cancer Hospital, Kunming, Yunnan, China; 2 Third Affiliated Hospital of Kunming Medical University, Kunming, Yunnan, China; 3 Peking University Cancer Hospital Yunnan, Kunming, Yunnan, China

**Keywords:** atherosclerosis, gut microbiota, probiotics, short-chain fatty acids, trimethylamine N-oxide, vascular function

## Abstract

**Background:**

Atherosclerosis (AS), the pathological basis of cardiovascular diseases, is a major cause of global mortality. Gut microbiota dysbiosis contributes to AS progression via trimethylamine N-oxide (TMAO), inflammation, and vascular dysfunction. Probiotics have emerged as a potential strategy against AS.

**Objective:**

To systematically review the mechanisms, efficacy, and future perspectives of probiotics in atherosclerosis.

**Methods:**

Preclinical and clinical evidence was summarized, focusing on gut microbiota, TMAO, short-chain fatty acids, lipid metabolism, inflammation, and vascular function.

**Results:**

Probiotics exert anti-atherosclerotic effects by restoring gut microbiota composition, reducing TMAO, increasing short-chain fatty acids, improving lipid profiles, alleviating inflammation, and protecting endothelial function. Clinical trials support beneficial effects on metabolic and vascular risk factors but are limited by small sample sizes and short durations.

**Conclusions:**

Probiotics are a promising and safe approach for AS intervention. Future large-scale, long-term trials and optimized probiotic designs are required to facilitate clinical application.

## 1 Introduction

Cardiovascular diseases (CVDs) account for 28.2% of global mortality, with atherosclerosis (AS) as their shared pathological hallmark (1). AS is a chronic inflammatory disorder characterized by subendothelial lipid accumulation, foam cell formation, and arterial plaque progression, driven by traditional risk factors (dyslipidemia, hypertension, diabetes) and non-traditional factors (gut microbiota dysbiosis, metabolic endotoxemia) (2, 3).

The human gut harbors ~10^14^ microorganisms, whose composition and function are tightly linked to host metabolism and immunity (4). Gut dysbiosis—marked by reduced α-diversity, increased pro-inflammatory bacteria (e.g., Enterobacteriaceae), and decreased beneficial bacteria (e.g., Lactobacillus, Bifidobacterium)—compromises intestinal barrier integrity, leading to translocation of lipopolysaccharide (LPS) and gut-derived uremic toxins (GDUTs) into the systemic circulation (5). These factors activate Toll-like receptor 4 (TLR4)-mediated inflammation and promote TMAO production, accelerating AS (6, 7).

Probiotics, primarily from the genera *Lactobacillus* and *Bifidobacterium*, have gained attention for their ability to restore gut homeostasis. Unlike pharmaceutical agents (e.g., statins) that carry side effects (e.g., myopathy), probiotics are generally safe and exert pleiotropic effects, including cholesterol lowering, anti-inflammation, and endothelial protection (8, 9). This review focuses on the role of probiotics in AS, integrating mechanisms, preclinical/clinical evidence, and future research needs.

## 2 PRISMA compliance and literature selection process


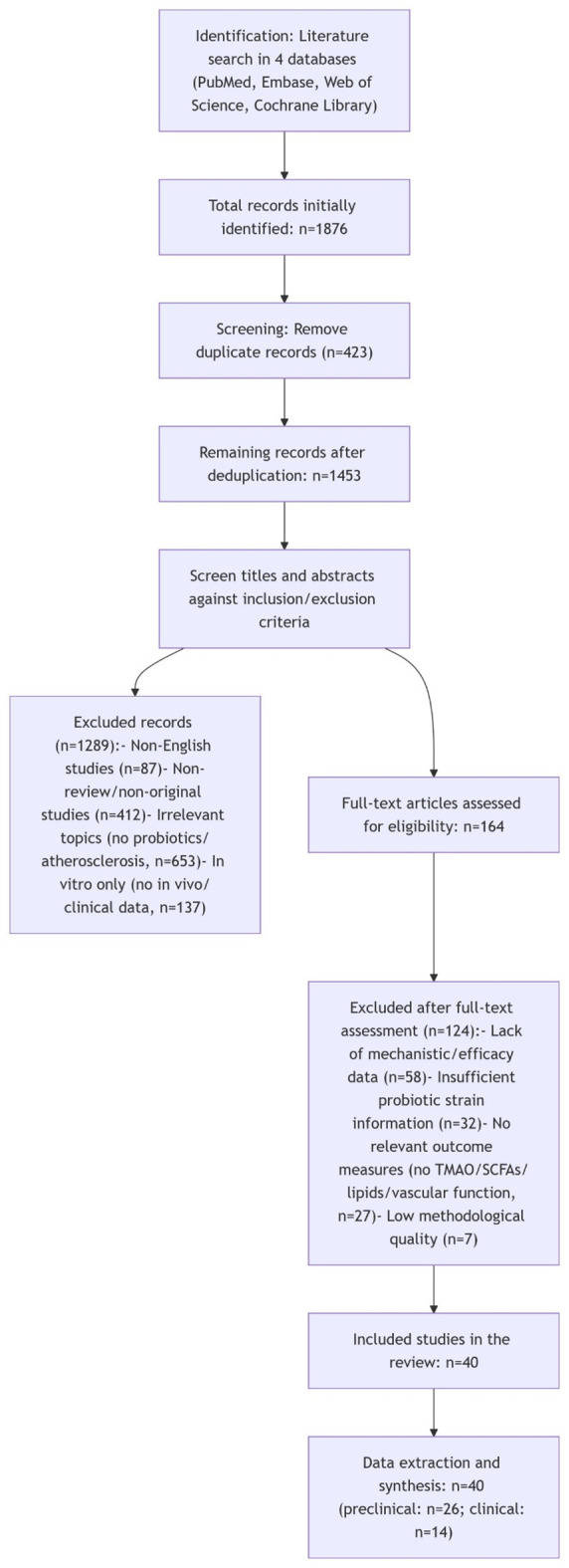


## 3 Gut microbiota dysbiosis and atherosclerosis: the mechanistic link

**Figure 1:**
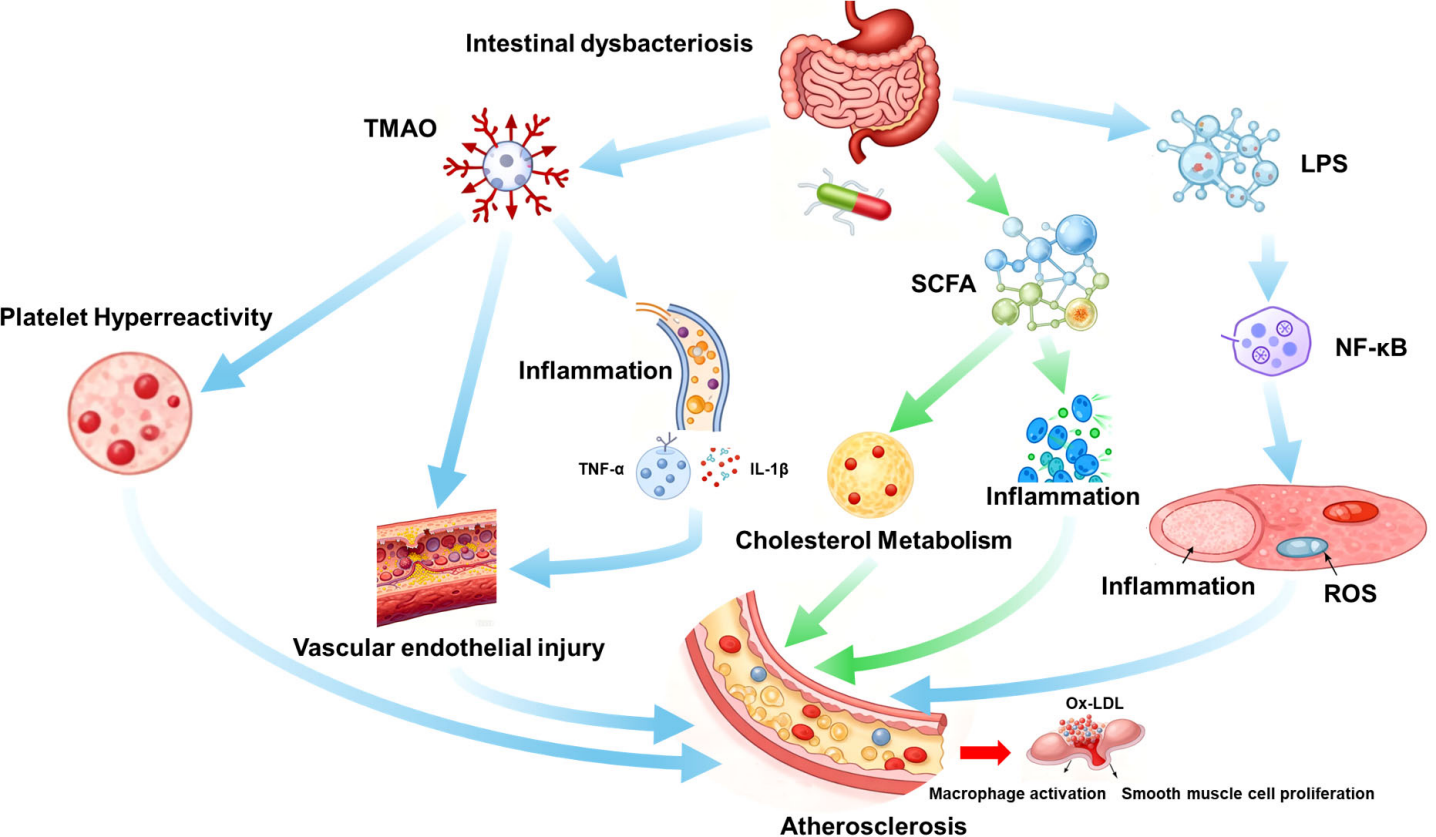
Schematic illustrating how gut dysbiosis promotes atherosclerosis via metabolic (TMAO/SCFAs), inflammatory (LPS/NF-κB), and vascular (endothelial dysfunction/oxidative stress) pathways. Blue arrows indicate pro-atherosclerotic effects; green arrows indicate protective effects. TMAO, trimethylamine N-oxide; SCFAs, short-chain fatty acids; LPS, lipopolysaccharide; NF-κB, nuclear factor κB; ROS, reactive oxygen species; ox-LDL, oxidized low-density lipoprotein.

Gut microbiota dysbiosis contributes to AS through three interconnected pathways: metabolic dysregulation, inflammatory activation, and vascular dysfunction (**Figure 1**).

### 3.1 Metabolic dysregulation: TMAO and short-chain fatty acids

#### 3.1.1 Trimethylamine N-oxide

Dietary choline, L-carnitine, and phosphatidylcholine (abundant in red meat, eggs) are metabolized by gut bacteria (e.g., *Clostridium*, *Enterococcus*) to trimethylamine (TMA), which is oxidized to TMAO by hepatic flavin monooxygenases (FMO3) (10). TMAO inhibits cholesterol efflux via downregulating ATP-binding cassette transporters (ABCA1/ABCG1), promotes platelet hyperreactivity, and enhances foam cell formation (11). In clinical studies, plasma TMAO levels >4 μM correlate with a 2.5-fold higher risk of myocardial infarction and stroke in patients with coronary artery disease (12).

#### 3.1.2 Short-chain fatty acids

Fermentation of dietary fiber by *Roseburia*, *Faecalibacterium prausnitzii*, and *Akkermansia muciniphila* produces acetate, propionate, and butyrate. SCFAs activate GPCR41/43 to reduce hepatic cholesterol synthesis, inhibit NF-κB-mediated inflammation, and enhance endothelial nitric oxide (NO) production (13). In ApoE^−^/^−^ mice, SCFAs reduce aortic plaque area by 30–40% (14).

### 3.2 Inflammatory activation: LPS and cytokine cascades

Gut barrier disruption (reduced occludin, ZO-1 expression) allows LPS translocation, activating TLR4/NF-κB signaling in macrophages and endothelial cells (15). This induces pro-inflammatory cytokines (TNF-α, IL-6, IL-1β) and adhesion molecules (VCAM-1, ICAM-1), promoting monocyte recruitment and foam cell formation (16). AS patients exhibit elevated serum LPS (endotoxemia) and C-reactive protein (CRP), which correlate with plaque instability (17).

### 3.3 Vascular dysfunction: endothelial injury and oxidative stress

Dysbiosis-derived metabolites (TMAO, LPS) and reduced SCFAs impair endothelial function by decreasing NO bioavailability and increasing reactive oxygen species (ROS) (18). Oxidized LDL (ox-LDL) accumulates in the subendothelium, triggering macrophage activation and smooth muscle cell proliferation—key steps in plaque formation (19).

## 4 Probiotics in atherosclerosis: mechanisms of action

Probiotics exert anti-atherosclerotic effects through strain-specific modulation of gut microbiota, metabolic homeostasis, inflammation, and vascular function (**Table 1**).

**Table 1:** Summary of key probiotic strains and their anti-atherosclerotic effects.

Strain(s)	Model/population	Key outcomes	Mechanisms	Reference
*L. plantarum ZDY04*	ApoE^−^/^−^ mice (choline-fed)	↓ TMAO (25%), ↓ aortic plaque (30%)	↑ *Lachnospiraceae*, ↓ TMA lyase activity	(22)
*B. longum* + *L. reuteri*	Stage III-IV CKD patients	↓ P-cresyl sulfate (18%), ↑ SCFAs	↑ BSH activity, ↓ gut permeability	(34)
*L. rhamnosus GG*	ApoE^−^/^−^ mice (HFD)	↓ LDL-C (22%), ↑ *Akkermansia*	↓ NPC1L1, ↑ cholesterol efflux	(20)
*L. plantarum 299v*	Men with stable CAD	↑ FMD (15%), ↓ IL-8 (20%)	↑ eNOS, ↓ NF-κB	(30)
*A. muciniphila*	LDLr^−^/^−^ mice (HFD)	↓ TMAO (28%), ↓ ox-LDL	↑ butyrate, ↓ oxidative stress	(25)

TMAO, trimethylamine N-oxide; CKD, chronic kidney disease; P-cresyl sulfate, p-cresyl sulfate; SCFAs, short-chain fatty acids; BSH, bile salt hydrolase; HFD, high-fat diet; CAD, coronary artery disease; FMD, flow-mediated dilation; eNOS, endothelial nitric oxide synthase; NF-κB, nuclear factor κB; ox-LDL, oxidized low-density lipoprotein.

### 4.1 Modulation of gut microbiota composition

#### 4.1.1 Restoring diversity

Probiotics increase α-diversity (Shannon/Simpson indices) and reverse (Firmicutes/Bacillota)/(Bacteroidetes/Bacteroidota) ratio imbalance. For example, *Lactobacillus rhamnosus GG* (LGG) increases *Lactobacillus* and *Akkermansia* abundance while reducing *Desulfovibrio* (a TMA-producing genus) in ApoE^−^/^−^ mice (20).

#### 4.1.2 Inhibiting pathogenic bacteria

*Bifidobacterium breve* and *Bifidobacterium longum* suppress *Enterobacteriaceae* and *Streptococcus* (pro-inflammatory genera) by competing for nutrients and producing bacteriocins (21).

### 4.2 Targeted regulation of metabolites

#### 4.2.1 Reducing TMAO

Strains like *L. plantarum ZDY04* and *Enterobacter aerogenes ZDY01* inhibit TMA lyase (a key enzyme in TMA synthesis) or downregulate hepatic FMO3, lowering serum TMAO by 20–30% in mice (22, 23). A clinical trial with *L. reuteri NCIMB 30242* showed a 15% reduction in plasma TMAO in hypercholesterolemic patients (24).

#### 4.2.2 Enhancing SCFAs

*Akkermansia muciniphila* and *Bifidobacterium animalis subsp. lactis* promote SCFA production by fermenting dietary fiber. In LDLr^−^/^−^ mice, *A. muciniphila* increases cecal butyrate levels by 45%, improving lipid metabolism (25).

### 4.3 Improving lipid profile

#### 4.3.1 Cholesterol lowering

Probiotics reduce intestinal cholesterol absorption via inhibiting Niemann-Pick C1-Like 1 (NPC1L1) and increasing bile salt hydrolase (BSH) activity. *L. acidophilus ATCC 4356* downregulates NPC1L1 in Caco-2 cells, reducing cholesterol absorption by 28% (26). *B. longum* increases fecal bile acid excretion, stimulating hepatic cholesterol conversion to bile acids (27).

#### 4.3.2 Regulating lipoproteins

Mixed probiotics (e.g., *L. plantarum* + *B. breve*) reduce LDL-C by 18–22% and increase HDL-C by 10–15% in ApoE^−^/^−^ mice and human trials (28, 29).

### 4.4 Alleviating inflammation and protecting endothelium

#### 4.4.1 Anti-inflammatory effects

Probiotics suppress NF-κB activation and reduce pro-inflammatory cytokines. *L. plantarum 299v* decreases serum IL-8 and TNF-α in patients with stable coronary artery disease (30). *B. bifidum* upregulates anti-inflammatory IL-10, inhibiting macrophage polarization to pro-inflammatory M1 phenotype (31).

#### 4.4.2 Endothelial protection

Probiotics enhance NO production and reduce oxidative stress. *Lactococcus lactis MG5125* increases endothelial NO synthase (eNOS) expression, improving flow-mediated dilation (FMD) in obese mice (32). *L. coryniformis CECT5711* reduces vascular ROS by activating antioxidant enzymes (SOD, GSH-Px) (33).

## 5 Preclinical and clinical evidence

### 5.1 Preclinical studies

Animal models (ApoE^−^/^−^, LDLr^−^/^−^ mice, rabbits) have consistently demonstrated probiotic efficacy:

#### 5.1.1 Plaque reduction

A 12-week intervention with *L. acidophilus ATCC 4356* reduced aortic plaque area by 40% in ApoE^−^/^−^ mice, associated with decreased intestinal cholesterol absorption (26).

#### 5.1.2 Metabolic regulation

*B. breve DSM 16604* decreased serum TMAO by 32% and increased propionate by 50% in choline-fed mice (21).

#### 5.1.3 Inflammation suppression

*L. fermentum CECT5716* reduced aortic TNF-α and VCAM-1 expression by 35% in HFD-fed rats (35).

### 5.2 Clinical trials

Clinical evidence, though limited by small sample sizes, supports probiotic benefits, Detailed characteristics of clinical trials investigating probiotics in atherosclerosis and related cardiovascular risk are summarized in **Table 2**:

**Table 2:** Clinical trials investigating probiotics and atherosclerosis/related cardiovascular risk.

Study (author, year)	Design	Population/sample size	Probiotic strain(s) and dose	Compared control	Outcomes measured	Key findings	Duration	Safety/adverse events	Risk of bias/notes
Jones et al., 2012 (24)	RCT, parallel	Hypercholesterolemic adults; n=127	Lactobacillus reuteri NCIMB 30242; 2.9×10^9^ CFU/capsule, 2×/day	Placebo (maltodextrin)	apoB-100, total cholesterol (TC), LDL-C, HDL-C, triglycerides (TG)	↓ LDL-C (11.64%), ↓ TC (9.14%); no change in HDL-C/TG	9 weeks	No serious adverse events; mild gastrointestinal discomfort (2 cases in probiotic group)	Low risk; small sample size; short duration
Ejtahed et al., 2011 (29)	RCT, parallel	Type 2 diabetes patients; n=60	Lactobacillus acidophilus La5 + Bifidobacterium lactis Bb12; 1×10^8^ CFU each strain/day (in yogurt)	Regular yogurt (without probiotics)	Serum TC, LDL-C, HDL-C, TG	↓ LDL-C (7.45%), ↓ TC (4.54%), no significant change in HDL-C	6 weeks	No adverse events reported	Moderate risk; lack of TMAO and vascular function measurements
Szulińska et al., 2018 (36)	RCT, parallel	Obese postmenopausal women; n=81	Multi-strain (Bifidobacterium bifidum W23, B. lactis W51/W52, L. acidophilus W37, L. brevis W63, L. casei W56, L. salivarius W24, Lactococcus lactis W19/W58); LD: 2.5×10^9^ CFU/day; HD: 1×10^10^ CFU/day	Placebo (maize starch and maltodextrins)	Systolic blood pressure (SBP), diastolic blood pressure (DBP), LDL-C, HDL-C, TC, TG,	↓ SBP, ↓ LDL-C no change in DBP/TG	12 weeks	Mild bloating (3 cases in probiotic group); no severe adverse events	Low risk; heterogeneous strain combination; limited to postmenopausal women
Tripolt et al., 2013 (37)	RCT, parallel	Metabolic syndrome patients; n=30	Lactobacillus casei Shirota; 3×6.5×10^9^ CFU/day (3×/day)	Standard medical therapy	insulin sensitivity (HOMA-IR), TC, LDL-C, sVCAM-1	↓ sVCAM-1; no significant change in HOMA-IR, lipids, or CRP	12 weeks	No adverse events reported	High risk; very small sample size; no vascular structure/function outcomes no placebo control
Poesen et al., 2016 (38)	RCT, parallel	Chronic kidney disease (CKD) patients (high AS risk); n=40	Prebiotic: Arabinoxylan oligosaccharides (AXOS) 10 g/day	Placebo (maltodextrin)	Serum p-cresyl sulfate (GDUT), TC, LDL-C, CRP, intestinal permeability	No significant change in p-cresyl sulfate; ↓ CRP (28%), ↓ intestinal permeability (15%);	4 weeks	No serious adverse events; mild diarrhea (2 cases in synbiotic group)	Moderate risk; prebiotic intervention (not probiotic); CKD-specific population
Malik et al., 2018 (30)	Nonrandomized pilot study	Men with stable coronary artery disease (CAD); n=20	Lactobacillus plantarum 299v; 2×10^10^ CFU/day	No control group (single-arm study)	Flow-mediated dilation (FMD), serum IL-8, TNF-α, TC, LDL-C	↑ FMD (33%), ↓ IL-8 (33%), ↓ TNF-α (18%); no significant change in TNF-α or lipids	6 weeks	No adverse events reported	Low risk; focused on endothelial function; limited to male CAD patients single-arm design

TMAO, trimethylamine N-oxide; LDL-C, low-density lipoprotein cholesterol; HDL-C, high-density lipoprotein cholesterol; CRP, C-reactive protein.

#### 5.2.1 Lipid improvement

A randomized controlled trial (RCT) of 81 obese postmenopausal women showed that a multi-strain probiotic (including *B. bifidum*, *L. acidophilus*) reduced systolic blood pressure (4 mmHg) and LDL-C (12%) (36).

#### 5.2.2 TMAO reduction

In 30 patients with metabolic syndrome, *L. casei Shirota* reduced plasma TMAO by 18% and improved insulin sensitivity (37).

#### 5.2.3 Renal-AS comorbidity

A synbiotic (probiotics + arabinoxylan oligosaccharides) reduced serum p-cresyl sulfate (a GDUT) by 22% in CKD patients, a population at high AS risk (38).

### 5.3 Limitations of current clinical studies

1. Small sample sizes (<100 participants in most trials).

2. Short follow-up (4–12 weeks), lacking long-term CVD outcome data.

3. Heterogeneity in probiotic strains, doses, and patient populations.

## 6 Future directions

To advance probiotic-based AS intervention, future research should address the following gaps:

### 6.1 Strain-specific optimization

1. Identify “AS-targeted” strains with validated mechanisms (e.g., TMAO reduction, SCFA production). For example, *L. plantarum* strains with high BSH activity or *A. muciniphila* variants with enhanced gut colonization.

2. Explore multi-strain combinations to synergize effects (e.g., *Lactobacillus* for lipid regulation + *Bifidobacterium* for anti-inflammation).

### 6.2 Clinical trial design

1. Conduct large-scale, long-term RCTs (>1,000 participants, 2–5 years) to assess hard endpoints (myocardial infarction, stroke).

2. Stratify patients by gut microbiota enterotypes or TMAO levels to identify responders.

### 6.3 Mechanistic deepening

1. Investigate probiotic-microbiota-host interactions using multi-omics (metagenomics, metabolomics, transcriptomics).

2. Explore epigenetic regulation (e.g., miRNA) by probiotics—for example, *L. acidophilus* modulates miR-155 (pro-inflammatory) and miR-21 (anti-apoptotic) in endothelial cells (39).

### 6.4 Formulation and delivery

1. Develop targeted delivery systems (e.g., enteric-coated capsules) to enhance probiotic survival in the gastrointestinal tract.

2. Combine probiotics with prebiotics (synbiotics) or phenolic compounds (e.g., quercetin, resveratrol) to boost efficacy—quercetin enhances *L. fermentum* survival and SCFA production (40).

## 7 Conclusions

Gut microbiota dysbiosis is a key driver of atherosclerosis, and probiotics offer a safe, accessible strategy for intervention. Through modulating gut microbiota, reducing pro-atherosclerotic metabolites (TMAO), enhancing SCFAs, and alleviating inflammation, probiotics show promise in preclinical models and early clinical trials. However, large-scale, long-term studies are needed to validate their efficacy in reducing CVD events. With optimized strains, formulations, and personalized approaches, probiotics could become a vital adjuvant therapy for atherosclerosis.

## Glossary

ABCA1: ATP-binding cassette transporter A1
ABCG1: ATP-binding cassette transporter G1
AS: Atherosclerosis
BSH: Bile salt hydrolase
CAD: Coronary artery disease
CKD: Chronic kidney disease
CRP: C-reactive protein
CVD: Cardiovascular disease
eNOS: Endothelial nitric oxide synthase
FMD: Flow-mediated dilation
FMO3: Flavin-containing monooxygenase 3
GPCR: G protein-coupled receptor
GDUT: Gut-derived uremic toxin
HFD: High-fat diet
HOMA-IR: Homeostatic model assessment of insulin resistance
ICAM-1: Intercellular adhesion molecule 1
IL: Interleukin
LPS: Lipopolysaccharide
LDL-C: Low-density lipoprotein cholesterol
HDL-C: High-density lipoprotein cholesterol
NF-кB: Nuclear factor kappa B
NO: Nitric oxide
NPC1L1: Niemann-Pick C1-like 1
ox-LDL: Oxidized low-density lipoprotein
RCT: Randomized controlled trial
ROS: Reactive oxygen species
SCFA: Short-chain fatty acid
SOD: Superoxide dismutase
GSH-Px: Glutathione peroxidase
TC: Total cholesterol
TG: Triglyceride
TLR4: Toll-like receptor 4
TNF-&alpha;: Tumor necrosis factor α
TMA: Trimethylamine
TMAO: Trimethylamine N-oxide
VCAM-1: Vascular cell adhesion molecule 1
ZO-1: Zonula occludens-1
